# Osteoporosis self-assessment tool for Asians and calcaneal quantitative ultrasound for identifying primary osteoporosis in Taiwanese postmenopausal women

**DOI:** 10.3389/fendo.2025.1639176

**Published:** 2025-09-12

**Authors:** Dung-Huan Liu, Chih-Sheng Lin, Pei-Ching Wu

**Affiliations:** ^1^ Department of Physical Medicine and Rehabilitation, China Medical University Hospital, Taichung, Taiwan; ^2^ Department of Physical Therapy, Graduate Institute of Rehabilitation Science, China Medical University, Taichung, Taiwan; ^3^ Graduate Degree Program of Biomedical Science and Engineering, National Yang Ming Chiao Tung University, Hsinchu, Taiwan; ^4^ Department of Biological Science and Technology, National Yang Ming Chiao Tung University, Hsinchu, Taiwan; ^5^ Center for Intelligent Drug Systems and Smart Bio-devices (IDS2B), National Yang Ming Chiao Tung University, Hsinchu, Taiwan; ^6^ Department of Chinese Medicine, China Medical University Hospital, Taichung, Taiwan; ^7^ College of Chinese Medicine, China Medical University, Taichung, Taiwan

**Keywords:** osteoporosis self-assessment tool for Asians (OSTA), calcaneal quantitative ultrasound (QUS), osteoporosis, postmenopausal women, dual-energy X-ray absorptiometry (DXA)

## Abstract

**Objectives:**

This study compares Osteoporosis Self-Assessment Tool for Asians (OSTA) and Calcaneal Quantitative Ultrasound (QUS) to detect primary osteoporosis among Taiwanese postmenopausal women and assess the consistency between both methods.

**Methods:**

8,883 postmenopausal women were selected from Taiwan Biobank. Osteoporosis was diagnosed using Dual-energy X-ray absorptiometry (DXA) with T-score≦-2.5 under WHO definition. QUS and OSTA were employed to assess osteoporosis risk, with statistical analyses including receiver operating characteristic curve (ROC) analysis, Delong’s test, and McNemar’s test to compare the performance of both tools. Youden’s J statistic identifies the optimal cut-off values of OSTA and QUS SI. Cohen’s kappa coefficient (k) and Spearman’s rank correlation coefficient (r_s_) assessed the correlation between OSTA, QUS, and DXA.

**Results:**

QUS outperformed OSTA with superior AUC in primary osteoporosis screening of Taiwanese postmenopausal women under WHO osteoporosis definition (AUC of QUS and OSTA are 0.737 and 0.703; p<0.05). They could independently screen and track the women at primary osteoporosis risk but not replace DXA for osteoporosis diagnosis, because they had a fair agreement of k (0.293~0.342) and a moderate correlation of r_s_ (0.424~0.481) with DXA. They couldn’t screen and track the women at primary osteoporosis risk interchangeably because their agreement is minimal (k=0.197; r_s_=0.271; p<0.05).

**Conclusions:**

QUS and OSTA are radiation-free, portable, less expensive and time-consuming, and effective screening tools for primary osteoporosis in Taiwanese postmenopausal women, with QUS being the superior method under WHO osteoporosis definition. After further age-stratified analysis for detecting primary osteoporosis in Taiwanese postmenopausal women, QUS outperformed OSTA in those aged 45 - 65, while OSTA outperformed QUS in those aged 66 - 80.

## 1 Introduction

Osteoporosis is the most common chronic metabolic bone disease, characterized by increased bone fragility, caused by various factors such as menopause, body weight, and aging. It affected people of all ages, genders, and races, especially Caucasians (white race), the elderly, and women. The global population of individuals aged 60 and older is projected to more than double by the year 2050, increasing from 962 million in 2017 to approximately 2.1 billion. By 2100, this demographic is expected to grow to over 3.1 billion, representing a more than threefold increase since 2017 (1). As the population ages and life expectancy increases, osteoporosis is becoming a global epidemic. Based on General Practice Research Database, the lifetime probability of experiencing an osteoporotic fracture in women aged 50 years has risen to 53.2% (2). Osteoporotic fractures are the most common and severe complication of osteoporosis, and the resulting high morbidity and mortality have imposed a massive healthcare burden on individuals, families, and society. It has been reported that by the year 2050, fractures caused by osteoporosis will have doubled and medical costs will skyrocket (3). Identifying people with osteoporosis risk and early interventions is the key to encumbering the progression of osteoporotic fractures, which can reduce hospital admissions, disabilities, mortality, and economic burdens to society (4).

Dual-energy X-ray absorptiometry (DXA) is the gold standard bone mineral density (BMD) examination for the diagnosis of osteoporosis (5). According to World Health Organization (WHO) definition, Osteoporosis is confirmed when a patient’s BMD value is 2.5 standard deviation (SD) lower than the reference value for young white female adults (T-score≦-2.5) (6). Considering the huge bulk, non-portability, professional operation, and high cost, DXA may not be suitable for widespread osteoporosis screening, especially in rural areas. In conjunction with the growth of the elderly population and the prevalence of osteoporosis, DXA will be more competitive. In Taiwan, DXA usage is mostly confined to hospitals, while most community health centers lack the equipment and cannot adequately serve the elderly population. A simple screening tool to detect the population with osteoporosis risk is necessary for clinicians in these areas.

Currently, simple, reliable, and cost-effective screening tools such as Calcaneal Quantitative Ultrasound (QUS) and Osteoporosis Self-Assessment Tool for Asians (OSTA) can quickly and easily identify people at risk of osteoporosis and fracture (7–10). According to WHO, neither OSTA nor QUS diagnoses osteoporosis, but they may have clinical benefits in prioritizing patients at high risk for DXA scanning. Furthermore, they may improve the screening efficiency by reducing the number of otherwise healthy individuals referred.

QUS serves as an alternative method for evaluating bone health and screening for osteoporosis by analyzing the propagation of ultrasonic waves through the calcaneus. The calcaneus is the primary skeletal site for QUS assessment, due to its high trabecular bone content and two lateral surfaces that facilitate ultrasound wave propagation and accessibility (11). These ultrasonic waves operate at frequencies beyond the normal human auditory range (greater than 20 kHz). Two main parameters generated by QUS are the speed of sound (SOS) and broadband ultrasound attenuation (BUA) (12). The SOS describes how fast sound waves propagate through various body structures. The BUA measures the loss of strength of ultrasound waves as they travel through soft tissue and bone. The Stiffness Index (SI) represents a composite metric that integrates SOS and BUA through various algorithmic approaches. The association of these variables with the QUS value was determined using proprietary software.

Compared to DXA, QUS is radiation-free, portable, less time-consuming, and less expensive, making it appropriate for research and clinical environments. The device manufacturers, study population (age, ethnicity, and gender), QUS measurement parameters, measured DXA and Achilles site have all influenced and differed in studies examining the discriminatory ability of QUS (13). As a result, QUS still lacks universal guidelines for distinguishing between normal and low BMD values.

Calcaneal QUS may aid in screening for osteoporosis, but there is no consensus on the device, variable, or cutoff value to use. The current evidence is insufficient to recommend any specific cutoff for reliably confirming or ruling out osteoporosis (14). Calcaneal QUS devices are effective in assessing fracture risk for certain populations, with the strongest evidence for Caucasian females over 55 years old, and fair evidence for Asian females above the same age (15). The GE Achilles Lunar QUS (GE Healthcare, Madison, WI) had great diagnosis accuracy with SI≦57 and a low chance of osteoporosis with SI>78, according to a study of older women who took part in the Epidemiology of Osteoporosis Study (15). Although several studies have compared values between calcaneal QUS and DXA, few studies have been conducted on Asian population (16–18). To our knowledge, it is possible to use QUS to prescreen Taiwanese with a high risk of osteoporosis based on its significant correlation with DXA and its optimal Youden’s index cutoff value of T-score for QUS to confirm osteoporosis is -2.725 (18).

Despite being less expensive than DXA, QUS devices are still costly and may not be available in all primary healthcare settings. Aside from QUS, other risk-based algorithms, such as OSTA, osteoporosis risk assessment tool (ORAI), simple calculated risk estimation (SCORE), and fracture risk assessment tool(FRAX ^®^), are now utilized to forecast osteoporosis and fragility fracture (19). The majority of these algorithms were created based on Caucasians. The OSTA was developed by Koh et al. using data from Asian women (8). Based on age and weight, the formula of OSTA is 0.2 × [body weight (Kg) - age (years)]. Patients with an OSTA score of ≦- 4 are considered high risk, those between -1 and -4 are ranged medium risk, and those of >-1 are ranged low risk of osteoporosis (8). According to the original study by Koh et al., the receiver operating characteristic (ROC) analysis using the highest Youden’s index (20) identified the optimal cut-off point of OSTA for predicting osteoporosis to be -1. This OSTA risk index of -1 demonstrated a sensitivity of 91% and a specificity of 45%, with an area under the curve of 0.79 (8).

Several studies have examined the agreement between QUS and OSTA in identifying people with osteoporosis. The above studies mostly compared osteoporosis screening tools under the osteoporosis diagnosis by QUS, which is rare by DXA. The main feature of this study is that the large-scale analysis study used DXA as the diagnostic benchmark for osteoporosis to compare the osteoporosis screening effectiveness of OSTA and QUS in Taiwanese postmenopausal women. Since OSTA does not require equipment, it could replace QUS as a free osteoporosis screening tool if the two methods are consistent. Therefore, this study hopes to evaluate the ability of OSTA and QUS to detect primary osteoporosis in Taiwanese postmenopausal women and the consistency between OSTA and QUS.

## 2 Materials and methods

### 2.1 Ethics statement

This study was approved by the Institutional Review Board (IRB) of China Medical University Hospital (CMUH110-REC2-065) and the Taiwan Biobank IRB (TWBR11008 - 02).

### 2.2 Data source, participants’ inclusion and exclusion criteria

#### 2.2.1 Data source

This study utilized data from the Taiwan Biobank, which (2021 - 06-21) includes approximately 179,623 participants aged 30 to 80 without a history of cancer. Taiwan Biobank contains health questionnaires, physical examination data, blood test results, and imaging data (including DXA and QUS). The same ISCD-certified technician conducted the BMD and SI measurements using the same DXA and QUS machines.

#### 2.2.2 Participants’ inclusion criteria

Taiwanese women who were selected from Taiwan Biobank and have been postmenopausal for twelve months or more.

#### 2.2.3 Participants’ exclusion criteria

Taiwanese women had missing QUS data, had missing DXA data at any one site (femoral neck, total hip, lumbar spine), had osteoporosis risk factors (including current drinking and smoking history, secondary osteoporosis disease, and long-term exposure to glucocorticoids), or had incomplete questionnaire data. Secondary osteoporosis disease includes type I (insulin-dependent) diabetes, osteogenesis imperfecta in adults, untreated long-standing hyperthyroidism, hypogonadism or premature menopause (<45 years), chronic malnutrition, malabsorption, and chronic liver disease.

### 2.3 Identify osteoporosis with QUS and OSTA

The study used the stiffness index (SI) of QUS data (Achilles InSight, GE, USA) to assess the osteoporosis risk of participants. In **Table 1**, the optimal cut-off values based on Youden’s J statistic (20) for QUS SI in predicting osteoporosis were identified as 79.5, 75.5, 74.5, and 77.5 for DXA-confirmed osteoporosis at any site (the lowest T-score measured at the DXA of the femoral neck, total hip, or lumbar spine), femoral neck, total hip, and lumbar spine, indicating a lower risk of osteoporosis for individuals with SI values exceeding these above optimal cut-off values. Notably, the study identified an optimal cut-off value of 79.5 for QUS SI under the WHO definition of osteoporosis, which is close to the 78 reported by Hans et al. (15); the optimal cut-off value of QUS SI for femoral neck DXA-determined osteoporosis was set at 75.5, which is similar to the 75.7 found by Kung et al. (17). Additionally, the study used the Osteoporosis Self-assessment Tool (OSTA) to evaluate the participants’ osteoporosis risk. The simplified formula for OSTA is 0.2 × [body weight (Kg) - age (years)], with a cut-off value of -1. Individuals with values above -1 were considered to have a lower risk of osteoporosis (8).

**Table 1:** Preformance of QUS and OSTA for primary osteoporosis screening in Taiwanese postmenopausal women with DXA-determined osteoporosis by different sites.

		DeLong's test			McNemar's test [sensitivity]		McNemar's test [specificity]					
	AUC	p-value	Cut-off point	TPR [sensitivity]	P-value	TNR [specificity]	P-value	FPR	FNR	PPV (precision)	NPV	Accuracy
Any one site												
SI	0.737	reference	79.500	0.686	reference	0.656	reference	0.343	0.314	0.683	0.660	0.672
OSTA	0.703	<0.05	-1.000	0.619	<0.05	0.674	0.070	0.326	0.381	0.672	0.622	0.646
Femoral neck												
SI	0.724	reference	75.500	0.635	reference	0.700	reference	0.300	0.365	0.455	0.830	0.682
OSTA	0.718	0.381	-1.000	0.707	<0.05	0.616	<0.05	0.388	0.293	0.418	0.841	0.639
OSTA	0.718	0.381	-1.190	0.671	<0.05	0.650	<0.05	0.350	0.329	0.431	0.833	0.656
Total hip												
SI	0.765	reference	74.500	0.734	reference	0.684	reference	0.316	0.266	0.233	0.952	0.690
OSTA	0.738	<0.05	-1.000	0.777	<0.05	0.566	<0.05	0.438	0.216	0.190	0.952	0.588
OSTA	0.738	<0.05	-1.630	0.681	<0.05	0.680	0.566	0.320	0.319	0.218	0.942	0.680
Lumbar spine												
SI	0.717	reference	77.500	0.629	reference	0.687	reference	0.313	0.371	0.629	0.687	0.661
OSTA	0.690	<0.05	-1.000	0.628	0.922	0.648	<0.05	0.352	0.372	0.601	0.674	0.639
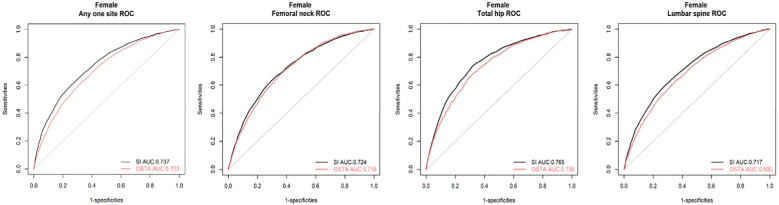												

AUC, Area Under Curve; Any one site, the lowest T-score at DXA of femoral neck, total hip or lumbar spine; SI, Stiffness Index of QUS; TPR, Ture Positive Rate; TNR, Ture Negative Rate; FPR, false positive rate; FNR, false negative rate; PPV, Positive Predictive Value; NPV, Negative Predictive Value; p<0.05 indicate statistically significant difference.

### 2.4 Diagnosis of osteoporosis

According to WHO definition, osteoporosis is diagnosed if the lowest T-score measured by DXA (DiscoveryTM QDRTM Bone Densitometry Systems (HOLOGIC) machine) at any one site of femoral neck, total hip, or lumbar spine (L1-L4) was less than or equal to -2.5 standard deviations (This study employed the young female Caucasian populations as the reference value according to WHO osteoporosis definition (21–23) and the DXA machine’s original setup).

### 2.5 Statistical analysis

This study used receiver operating characteristic (ROC) curve analysis and Delong’s test to evaluate the ability of OSTA and QUS for osteoporosis risk screening and compare the area under the curve (AUC) of both tools. The highest Youden’s index identifies the optimal cut-off values of OSTA and QUS SI; Youden’s J statistic is calculated using the formula: J=Sensitivity+Specificity-1 (20).The sensitivity was defined as the proportion of women diagnosed with osteoporosis (T-scores ≤ -2.5) who had a positive test (i.e., index values below the cut-off). The specificity was defined as the proportion of women diagnosed without osteoporosis who tested normal (i.e., having index values above or equal to the cut-off). McNemar’s test compared both tools’ sensitivity, specificity, and negative predictive value (NPV). Cohen’s kappa coefficient (k) and Spearman’s rank correlation coefficient (r_s_) assessed the correlation between OSTA, QUS, and DXA. All analyses were performed using R version 4.3.2 (http://www.R-project.org). A p-value of <0.05 indicated a statistically significant difference.

## 3 Result

### 3.1 Study population selection

**Figure 1:**
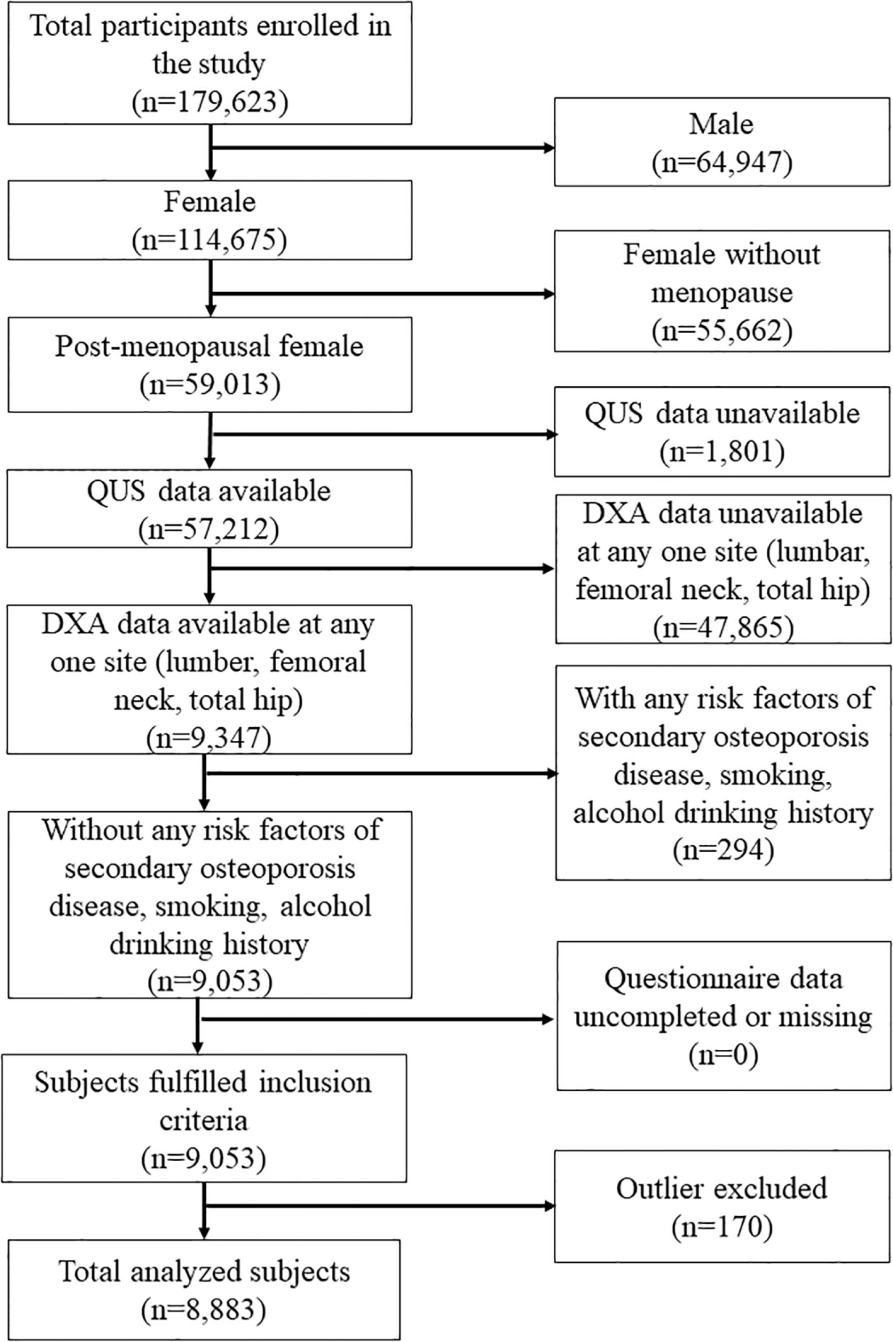
The flowchart of the study.

Following the flowchart of **Figure 1**, 114,675 women were enrolled in this study. The study excluded women who were not postmenopausal, had missing QUS data, had missing DXA data at any one site (femoral neck, total hip, lumbar spine), had osteoporosis risk factors (including current drinking and smoking history, secondary osteoporosis disease, and long-term exposure to glucocorticoids), or had incomplete questionnaire data. Secondary osteoporosis disease includes type I (insulin-dependent) diabetes, osteogenesis imperfecta in adults, untreated long-standing hyperthyroidism, hypogonadism or premature menopause (<45 years), chronic malnutrition, malabsorption, and chronic liver disease. Finally, 8883 women were included and analyzed after excluding outliers.

### 3.2 Characteristics of the study population

In **Table 2**, the subjects were categorized into two groups according to the WHO criteria’s diagnosis of osteoporosis (T-score ≤ -2.5): 4609 (51.89%) with osteoporosis and 4274 (48.11%) without osteoporosis. All characteristics were significantly different between both groups (p<0.05).

**Table 2:** Baseline characteristics of all subjects in the study.

	Postmenopausal women		
	T score ≦ -2.5	-2.5 < T score	
Total N=8883	N=4609	N=4274	P-value
**Age (years)** mean,(SD^1^)	62.39 (5.50)	59.99 (5.96)	<0.05
**Weight (kg)** mean,(SD^1^)	54.85 (7.66)	59.66 (8.15)	<0.05
[min, max]	[34.4, 84.2]		
**Height (cm)** mean,(SD^1^)	154.83 (5.39)	156.51 (5.20)	<0.05
[min, max]	[123.5, 177.5]		
**BMI (kg/m^2^)** mean,(SD^1^)	22.89 (3.04)	24.37 (3.22)	<0.05
T-score of DXA			
Any one site^2^ mean,(SD^1^)	-3.28 (0.63)	-1.63 (0.63)	<0.05
Femoral neck mean,(SD^1^)	-2.46 (0.67)	-1.32 (0.72)	<0.05
Total hip mean,(SD^1^)	-1.94 (0.71)	-0.78 (0.74)	<0.05
Lumbar spine mean,(SD^1^)	-3.13 (0.78)	-1.28 (0.87)	<0.05
**Stiffness Index of QUS** mean,(SD^1^)	74.94 (11.79)	86.02 (13.59)	<0.05
**OSTA Index value** mean,(SD^1^)	-1.51 (1.87)	-0.07 (1.98)	<0.05

SD^1^, Any one site^2^

^1^SD, standard deviation; p<0.05 indicate statistically significant difference.

^2^Any one site: the lowest T-score at DXA of femoral neck, total hip, or lumbar spine.

### 3.3 The assessment of OSTA and QUS in detecting osteoporosis

In **Table 1**, the evaluation of osteoporosis screening tools based on WHO osteoporosis definition, the AUC, sensitivity, specificity, and NPV were 0.737, 0.686, 0.656, and 0.660 for QUS with an optimal cut-off value of 79.5; 0.703, 0.619, 0.674 and 0.622 for OSTA with an optimal cut-off value of -1(the same cut-off value of OSTA as Koh et al.). Under the cut-off values of 79.5 and -1 for QUS and OSTA, the AUC, sensitivity, and NPV of QUS significantly outperformed OSTA (p<0.05). The specificity of QUS was mildly more than OSTA without a significant difference (p>0.05). The capability of QUS to detect osteoporosis outperformed OSTA, with superior AUC, sensitivity, NPV, and comparable specificity.

For femoral neck DXA-determined osteoporosis with T-score ≤ -2.5, the AUC, sensitivity, specificity, and NPV were 0.724, 0.635, 0.700, and 0.830 for QUS with an optimal cut-off value of 75.5; 0.718, 0.671, 0.650, and 0.833 for OSTA with an optimal cut-off value of -1.19; 0.718, 0.707, 0.616, and 0.841 for OSTA with cut-off value of -1. Under the cut-off values of 75.5 and -1 for QUS and OSTA, the AUC of QUS was mildly more than OSTA without a significant difference (p>0.05). The sensitivity and NPV of OSTA were significantly better than QUS (p<0.05). The specificity of QUS significantly outperformed OSTA (p>0.05). The capability of OSTA to detect osteoporosis was comparable to QUS, with comparable AUC, superior sensitivity and NPV, and inferior specificity.

For total hip DXA-determined osteoporosis with T-score ≤ -2.5, the AUC, sensitivity, specificity, and NPV were 0.765, 0.734, 0.684, and 0.952 for QUS with an optimal cut-off value of 74.5; 0.738, 0.681, 0.680, and 0.942 for OSTA with an optimal cut-off value -1.63; 0.738, 0.777, 0.566, and 0.952 for OSTA with a cut-off value -1. Under the cut-off values of 74.5 and -1 for QUS and OSTA, the AUC and specificity of QUS were significantly better than OSTA (p<0.05). The sensitivity of OSTA significantly outperformed QUS (p<0.05). The NPV of OSTA was the same as QUS (p>0.05). The capability of QUS to detect osteoporosis outperformed OSTA, with superior AUC and specificity, inferior sensitivity, and the same NPV.

For lumbar DXA-determined osteoporosis with T-score ≤ -2.5, the AUC, sensitivity, specificity, and NPV were 0.717, 0.629, 0.687, and 0.687 for QUS with an optimal cut-off value of 77.5; 0.690, 0.628, 0.648, and 0.674 for OSTA with an optimal cut-off value of -1(the same cut-off value of OSTA as Koh et al.). Under the cut-off values of 77.5 and -1 for QUS and OSTA, the AUC, specificity, and NPV of QUS significantly outperformed OSTA (p<0.05). The sensitivity of QUS was the same as OSTA (p>0.05). The capability of QUS to detect osteoporosis outperformed OSTA, with superior AUC, specificity, NPV, and the same sensitivity.

In summary, QUS outperformed OSTA with significantly superior AUC, sensitivity, and NPV (all p<0.05) under WHO osteoporosis definition. For DXA-determined osteoporosis of the total hip or lumbar spine with T-score ≤ -2.5, QUS outperformed OSTA with significantly superior AUC and specificity (all p < 0.05). For femoral neck DXA-determined osteoporosis with T-score ≤ -2.5, the capability of OSTA to detect primary osteoporosis was comparable to QUS with comparable AUC (p>0.05), superior sensitivity (p<0.05), and superior NPV (p<0.05).

In brief, whether osteoporosis is diagnosed according to WHO definition, total hip, or lumbar spine DXA T-score ≤ -2.5, QUS outperformed OSTA with superior AUC in detecting primary osteoporosis of Taiwanese postmenopausal women (p<0.05). However, the capability of OSTA to detect primary osteoporosis was comparable to QUS with comparable AUC for femoral neck DXA-determined osteoporosis with T-score ≤ -2.5 (p>0.05).

### 3.4 Comparison between OSTA and QUS at each age stratification

**Tables 3**–**6** divided the participants into two age groups: 45 - 65 and 66 - 80. Under WHO osteoporosis definition in **Table 3**, the sensitivity, specificity, and NPV were 0.646, 0.693, and 0.686 for QUS (cut-off value: 79.5) in the aged 45 - 65; 0.485, 0.774, and 0.627 for OSTA (cut-off value: -1) in the aged 45 - 65; 0.754, 0.543, and 0.573 for QUS in the aged 66 - 80; 0.850, 0.360, and 0.593 for OSTA in the aged 66 - 80. At the age of 45 - 65, the sensitivity and NPV of QUS outperformed OSTA (p<0.05), with inferior specificity (p<0.05). The capability of QUS to detect osteoporosis outperformed OSTA. At the age of 66 - 80, the sensitivity of OSTA outperformed QUS (p<0.05), with inferior specificity (p<0.05) and mildly superior NPV (p>0.05). The capability of OSTA to detect osteoporosis outperformed QUS.

**Table 3:** Comparison between QUS and OSTA for osteoporosis screening in Taiwanese postmenopausal women with DXA-determined osteoporosis by any one site (The lowest T-score at DXA of femoral neck, total hip or lumbar spine).

Female	BMD		Total	Sensitivity		Specificity		NPV	
	T score ≦ -2.5	-2.5 < T score		%	95% CI	%	95% CI	%	95% CI
All age									
SI value									
High risk(≦79.5)	3160	1467	4627	0.686	0.672-0.699	0.657	0.643-0.671	0.660	0.645-0.674
Low risk (>79.5)	1449	2807	4256						
Total	4609	4274	8883						
McNemar's Test p-value				reference		reference		reference	
OSTA value									
High risk (≦-1)	2855	1393	4248	0.619	0.605-0.633	0.674	0.660-0.688	0.622	0.608-0.636
Low risk (>-1)	1754	2881	4635						
Total	4609	4274	8883						
McNemar's Test p-value				<0.05		0.070		<0.05	
45-65 y/o									
SI value									
High risk(≦79.5)	1877	997	2874	0.646	0.628-0.663	0.693	0.677-0.709	0.686	0.670-0.702
Low risk (>79.5)	1030	2249	3279						
Total	2907	3246	6153						
McNemar's Test p-value				reference		reference		reference	
OSTA value									
High risk (≦-1)	1409	733	2142	0.485	0.467-0.503	0.774	0.760-0.789	0.627	0.612-0.641
Low risk (>-1)	1498	2513	4011						
Total	2907	3246	6153						
McNemar's Test p-value				<0.05		<0.05		<0.05	
65-80 y/o									
SI value									
High risk(≦79.5)	1286	474	1760	0.754	0.733-0.775	0.543	0.512-0.572	0.573	0.548-0.599
Low risk (>79.5)	420	563	983						
Total	1706	1037	2743						
McNemar's Test p-value				reference		reference		reference	
OSTA value									
High risk (≦-1)	1450	664	2114	0.850	0.833-0.867	0.360	0.331-0.390	0.593	0.560-0.627
Low risk (>-1)	256	373	629						
Total	1706	1037	2743						
McNemar's Test p-value				<0.05		<0.05		0.374	

( ):The Parentheses mark refers to the cut-off value of SI or OSTA; SI, Stiffness Index of QUS; BMD, Bone Marrow Density; CI, Confidence Interval; NPV, Negtive Predictive Value; p<0.05 indicate statistically significant difference.

**Table 4:** Comparison between QUS and OSTA for osteoporosis screening in Taiwanese postmenopausal women with DXA-determined osteoporosis by lumbar spine.

Female	BMD		Total		Sensitivity		Specificity		NPV			
	T score ≦ -2.5	-2.5 < T score			%	95% CI	%	95% CI	%	95% CI
All age												
SI value												
High risk(≦77.5)	2558	1507	4065		0.629	0.615-0.644	0.687	0.674-0.700	0.687	0.674-0.701
Low risk (>77.5)	1506	3312	4818							
Total	4064	4819	8883							
McNemar's Test p-value					reference		reference		reference	
OSTA value												
High risk (≦-1)	2554	1694	4248		0.628	0.614-0.643	0.648	0.635-0.662	0.674	0.661-0.688
Low risk (>-1)	1510	3125	4635							
Total	4064	4819	8883							
McNemar's Test p-value					0.922		<0.05		<0.05	
45-65 y												
SI value												
High risk(≦77.5)	1499	990	2489		0.584	0.565-0.603	0.724	0.709-0.739	0.709	0.694-0.724	
Low risk (>77.5)	1066	2598	3664								
Total	2565	3588	6153								
McNemar's Test p-value					reference		reference		reference		
OSTA value												
High risk (≦-1)	1266	876	2142		0.493	0.473-0.513	0.756	0.743-0.769	0.676	0.667-0.686	
Low risk (>-1)	1299	2712	4011								
Total	2565	3588	6153								
McNemar's Test p-value					<0.05		<0.05		<0.05		
65-80 y												
SI value												
High risk(≦77.5)	1059	517	1576		0.706	0.683-0.730	0.58	0.552-0.608	0.619	0.591-0.647	
Low risk (>77.5)	440	714	1154								
Total	1499	1231	2730								
McNemar's Test p-value						reference		reference		reference		
OSTA value												
High risk (≦-1)	1288	818	2106		0.859	0.842-0.877	0.335	0.310-0.362	0.662	0.629-0.697	
Low risk (>-1)	211	413	624								
Total	1499	1231	2730								
McNemar's Test p-value					<0.05		<0.05		<0.05		

( )The Parentheses mark refers to the cut-off value of SI or OSTA; SI, Stiffness Index of QUS; BMD, Bone Marrow Density; CI, Confidence Interval; NPV, Negtive Predictive Value; p<0.05 indicate statistically significant difference.

**Table 5:** Comparison between QUS and OSTA for osteoporosis screening in Taiwanese postmenopausal women with DXA-determined osteoporosis by femoral neck.

Female	BMD		Total		Sensitivity		Specificity		NPV			
	T score ≦ -2.5	-2.5 < T score			%	95% CI	%	95% CI	%	95% CI		
All age												
SI value												
High risk(≦75.5)	1597	1912	3509		0.635	0.617-0.654	0.700	0.689-0.711	0.830	0.819-0.840
Low risk (>75.5)	916	4458	5374							
Total	2513	6370	8883							
McNemar's Test p-value					reference		reference		reference	
OSTA value												
High risk (≦-1)	1777	2471	4248		0.707	0.689-0.725	0.612	0.600-0.624	0.841	0.831-0.852
Low risk (>-1)	736	3899	4635							
Total	2513	6370	8883							
McNemar's Test p-value					<0.05		<0.05		<0.05	
High risk (≦-1.19)	1685	2228	3913		0.671	0.652-0.689	0.650	0.639-0.662	0.833	0.823-0.844
Low risk (>-1.19)	828	4142	4970							
Total	2513	6370	8883							
McNemar's Test p-value					<0.05		<0.05		0.475	
45-65 y												
SI value												
High risk(≦75.5)	885	1229	2114		0.588	0.563-0.613	0.736	0.723-0.748	0.846	0.835-0.857	
Low risk (>75.5)	621	3418	4039								
Total	1506	4647	6153								
McNemar's Test p-value					reference		reference		reference		
OSTA value												
High risk (≦-1)	885	1257	2142		0.588	0.563-0.613	0.730	0.717-0.742	0.845	0.834-0.856	
Low risk (>-1)	621	3390	4011								
Total	1506	4647	6153								
McNemar's Test p-value					>0.999		0.494		0.847		
High risk (≦-1.19)	813	1079	1892		0.540	0.515-0.565	0.768	0.756-0.780	0.837	0.826-0.848	
Low risk (>-1.19)	693	3568	4261								
Total	1506	4647	6153								
McNemar's Test p-value					<0.05		<0.05		0.101		
65-80 y												
SI value												
High risk(≦75.5)	712	683	1395		0.707	0.679-0.735	0.604	0.581-0.627	0.779	0.757-0.801	
Low risk (>75.5)	295	1040	1335								
Total	1007	1723	2730								
McNemar's Test p-value					reference			reference		reference		
OSTA value												
High risk (≦-1)	892	1214	2106		0.886	0.866-0.905	0.295	0.274-0.317	0.816	0.785-0.846	
Low risk (>-1)	115	509	624								
Total	1007	1723	2730								
McNemar's Test p-value					<0.05		<0.05		<0.05		
High risk (≦-1.19)	872	1149	2021		0.866	0.845-0.887	0.333	0.311-0.355	0.810	0.781-0.838	
Low risk (>-1.19)	135	574	709								
Total	1007	1723	2730								
McNemar's Test p-value					<0.05		<0.05		<0.055		

( )The Parentheses mark refers to the cut-off value of SI or OSTA; SI, Stiffness Index of QUS; BMD, Bone Marrow Density; CI, Confidence Interval; NPV, Negtive Predictive Value; p<0.05 indicate statistically significant difference.

**Table 6:** Comparison between QUS and OSTA for osteoporosis screening in Taiwanese postmenopausal women with DXA-determined osteoporosis by total hip.

Female	BMD		Total		Sensitivity		Specificity		NPV			
	T score ≦ -2.5	-2.5 < T score			%	95% CI	%	95% CI	%	95% CI		
All age												
SI value												
High risk(≦74.5)	755	2484	3239		0.734	0.707-0.761	0.684	0.673-0.694	0.952	0.946-0.957
Low risk (>74.5)	273	5371	5644							
Total	1028	7855	8883							
McNemar's Test p-value					reference		reference		reference	
OSTA value												
High risk (≦-1)	806	3442	4248		0.784	0.759-0.809	0.562	0.551-0.573	0.952	0.946-0.958
Low risk (>-1)	222	4413	4635							
Total	1028	7855	8883							
McNemar's Test p-value					<0.05		<0.05		0.892	
High risk (≦-1.63)	700	2515	3215		0.681	0.652-0.709	0.680	0.670-0.690	0.942	0.936-0.948
Low risk (>-1.63)	328	5340	5668							
Total	1028	7855	8883							
McNemar's Test p-value					<0.05		0.566		<0.05	
45-65 y												
SI value												
High risk(≦74.5)	419	1503	1922		0.688	0.651-0.725	0.729	0.717-0.741	0.955	0.949-0.961	
Low risk (>74.5)	190	4041	4231								
Total	609	5544	6153								
McNemar's Test p-value					reference		reference		reference		
OSTA value												
High risk (≦-1)	406	1736	2142		0.667	0.629-0.704	0.687	0.675-0.699	0.949	0.943-0.956	
Low risk (>-1)	203	3808	4011								
Total	609	5544	6153								
McNemar's Test p-value					0.410		<0.05		0.123		
High risk (≦-1.63)	322	1078	1400		0.529	0.489-0.568	0.806	0.795-0.816	0.940	0.933-0.946	
Low risk (>-1.63)	287	4466	4753								
Total	609	5544	6153								
McNemar's Test p-value					<0.05		<0.05		<0.05		
65-80 y												
SI value												
High risk(≦74.5)	336	981	1317		0.802	0.764-0.840	0.576	0.555-0.596	0.941	0.929-0.954	
Low risk (>74.5)	83	1330	1413								
Total	419	2311	2730								
McNemar's Test p-value					reference			reference		reference		
OSTA value												
High risk (≦-1)	400	1706	2106		0.955	0.935-0.975	0.262	0.244-0.280	0.970	0.956-0.983	
Low risk (>-1)	19	605	624								
Total	419	2311	2730								
McNemar's Test p-value					<0.05		<0.05		<0.05		
High risk (≦-1.63)	378	1437	1815		0.902	0.874-0.931	0.378	0.358-0.398	0.955	0.942-0.969	
Low risk (>-1.63)	41	874	915								
Total	419	2311	2730								
McNemar's Test p-value					<0.05		<0.05		0.095		

( )The Parentheses mark refers to the cut-off value of SI or OSTA; SI, Stiffness Index of QUS; BMD, Bone Marrow Density; CI, Confidence Interval; NPV, Negtive Predictive Value; p<0.05 indicate statistically significant difference.

Under the diagnosis of osteoporosis based on the T-score≦ -2.5 at the lumbar spine in **Table 4**, the sensitivity, specificity, and NPV were 0.584, 0.724, and 0.709 for QUS (cut-off value: 77.5) in the aged 45 - 65; 0.493, 0.756, and 0.676 for OSTA (cut-off value: -1) in the aged 45 - 65; 0.706, 0.580, and 0.619 for QUS in the aged 66 - 80; 0.859, 0.335, and 0.662 for OSTA in the age 66 - 80. At the age of 45 - 65, the sensitivity and NPV of QUS outperformed OSTA (p<0.05), with inferior specificity (p<0.05). The capability of QUS to detect osteoporosis outperformed OSTA. At the age of 66 - 80, the sensitivity and NPV of OSTA outperformed QUS (p<0.05), with inferior specificity (p<0.05). The capability of OSTA to detect osteoporosis outperformed QUS.

Under the diagnosis of osteoporosis based on the T-score ≦ -2.5 at the femoral neck in **Table 5**, the sensitivity, specificity, and NPV were 0.588, 0.736, and 0.846 for QUS (cut-off value: 75.5) in the aged 45 - 65; 0.588, 0.730, and 0.845 for OSTA (cut-off value: -1) in the aged 45 - 65; 0.707, 0.604, and 0.779 for QUS in the aged 66 - 80; 0.886, 0.295, and 0.816 for OSTA in the aged 66 - 80. At the age of 45 - 65, the sensitivity of QUS was the same as the OSTA, with mildly superior specificity and NPV(p>0.05). The capability of QUS to detect osteoporosis outperformed OSTA. At the age of 66 - 80, the sensitivity and NPV of OSTA outperformed QUS (p<0.05), with inferior specificity (p<0.05). The capability of OSTA to detect osteoporosis outperformed QUS.

Under the diagnosis of osteoporosis based on the T-score ≦ -2.5 at the total hip in **Table 6**, the sensitivity, specificity, and NPV were 0.688, 0.729, and 0.955 for QUS (cut-off value: 74.5) in the aged 45 - 65; 0.667, 0.687, and 0.949 for OSTA (cut-off value: -1) in the aged 45 - 65; 0.802, 0.576, and 0.941 for QUS in the aged 66 - 80; 0.955, 0.262, and 0.970 for OSTA in the aged 66 - 80. At the age of 45 - 65, the specificity of QUS outperformed OSTA (p<0.05), with mildly superior sensitivity and NPV (p>0.05). The capability of QUS to detect osteoporosis outperformed OSTA. At the age of 66 - 80, the sensitivity and NPV of OSTA outperformed QUS (p<0.05), with inferior specificity (p<0.05). The capability of OSTA to detect osteoporosis outperformed QUS.

In summary, after further age-stratified analysis for detecting primary osteoporosis in Taiwanese postmenopausal women, whether osteoporosis is diagnosed according to WHO definition, femoral neck, total hip, or lumbar spine DXA T-score ≤ -2.5, QUS outperformed OSTA with superior sensitivity and NPV in those aged 45 - 65 (all p<0.05, except for femoral neck and total hip with p>0.05), while OSTA outperformed QUS with superior sensitivity and NPV in those aged 66 - 80 (all p<0.05, except NPV under WHO definition with p>0.05).

## 4 Discussion

Under WHO osteoporosis definition, QUS (AUC: 0.737, with a cut-off value of SI: 79.5) and OSTA (AUC: 0.703, with a cut-off value: -1) are good and sufficient primary osteoporosis screening tools for postmenopausal Taiwanese women with AUCs over 0.7 in **Table 1**. The AUC of the diagnostic tool <0.7 is considered unacceptable (24). In **Table 7**, the k between OSTA and DXA or between QUS and DXA are 0.293 or 0.342 (p<0.05); the r_s_ between OSTA and DXA or between QUS and DXA are 0.424 or 0.481 (p<0.05). They had a fair agreement of k and a moderate correlation of r_s_ with DXA. This means they could independently screen and track the women at primary osteoporosis risk but not replace DXA for osteoporosis diagnosis. The agreement between QUS and OSTA was limited despite a statistically significant correlation (k=0.197, slight agreement; r_s_=0.271, weak degree; all p<0.05). In other words, they couldn’t screen and track the women at primary osteoporosis risk interchangeably because their agreement is minimal. These results are similar to the previous studies on Chinese women (k=0.151, slight agreement; r_s_=0.418, moderate degree; all p<0.001) (25) and Taiwanese women (r_s_=0.200, weak degree; p<0.05) (16). Chin et al. also found similar results with slight to fair agreement of k and weak degree of r_s_ between QUS and OSTA in Chinese (k=0.186, r_s_=0.325; all p<0.05), Malay (k=0.338, r_s_=0.348; all p<0.05), and Indian women (k=0.235, r_s_=0.345; all p>0.05) (26).

Table 7The agreement and correlation between OSTA, QUS and DXA T-score at different sites.

**Table:** 

	Cohen's kappa	p-value	Spearman's correlation	p-value
Correlation between OSTA and QUS				
OSTA and SI			0.271	<0.05
OSTA(-1) and SI	0.197	<0.05		
Correlation between OSTA and DXA				
OSTA and T-score of Any one site DXA			0.424	<0.05
OSTA(-1) and T-score	0.293	<0.05		
OSTA and T-score of Femoral neck DXA			0.444	<0.05
OSTA(-1.19) and T-score	0.274	<0.05		
OSTA(-1) and T-score	0.264	<0.05		
OSTA and T-score of Total hip DXA			0.446	<0.05
OSTA(-1.63) and T-score	0.272	<0.05		
OSTA(-1) and T-score	0.146	<0.05		
OSTA and T-score of Lumbar spine DXA			0.389	<0.05
OSTA(-1) and T-score	0.276	<0.05		
Correlation between QUS and DXA				
SI and T-score of Any one site DXA			0.481	<0.05
SI(79.5) and T-score	0.342	<0.05		
SI and T-score of Femoral neck DXA			0.464	<0.05
SI(75.5) and T-score	0.273	<0.05		
SI and T-score of Total hip DXA			0.442	<0.05
SI(74.5) and T-score	0.317	<0.05		
SI and T-score of Lumbar spine DXA			0.492	<0.05
SI(77.5) and T-score	0.171	<0.05

**Table:** 

Cohen’s kappa	Interpretation
0	no agreement
0.1-0.2	slight agreement
0.21-0.4	fair agreement
0.41-0.6	moderate agreement
0.61-0.80	substantial agreement
0.81-0.99	near prefect agreement
1	prefect agreement

**Table:** 

Spearman's correlation	degree
0	no correlation
0-0.19	very weak
0.2-0.39	weak
0.40-0.59	moderate
0.60-0.79	strong
0.80-1.00	very strong

( )The Parentheses mark refers to the cut-off value of SI or OSTA; SI, Stiffness Index of QUS.

T-score of Any one site DXA, the lowest T-score of femoral neck, total hip or lumbar spine DXA; p<0.05 indicate statistically significant correlation.

The k and r_s_ between QUS and DXA were better than those between OSTA and DXA under WHO osteoporosis definition (p<0.05) in **Table 7**. This means QUS rather than OSTA had a stronger correlation with DXA. After comparing AUCs, sensitivity, and NPV by Delong’s test and McNemar’s test, QUS outperformed OSTA with superior AUC, sensitivity, and NPV in primary osteoporosis screening of Taiwanese postmenopausal women under WHO osteoporosis definition (AUC, sensitivity, NPV of QUS and OSTA are 0.737, 68.6%, 66.0%, and 0.703, 61.9%, 62.2%; p<0.05). According to the Kung AW et al. study in Hong Kong, OSTA had better AUC, sensitivity, NPV, and r_s_ with femoral neck DXA than QUS in primary osteoporosis screening of postmenopausal women for femoral neck DXA-determined osteoporosis (AUC, sensitivity, NPV, and r_s_ of OSTA and QUS are 0.80, 88.0%, 94.2%, 0.62 and 0.78, 81.0%, 92.7%, 0.36; the cut-off value of OSTA and QUS SI are -1 and 75.7). However, the capability of OSTA to detect osteoporosis was comparable to QUS with no statistical difference in the AUC comparison between OSTA and QUS (P>0.05) (17). The above result of Kung AW et al. study was similar to this study, in that the capability of OSTA to detect osteoporosis was comparable to QUS, with comparable AUC, superior sensitivity and NPV, and inferior r_s_ with femoral neck BMD in postmenopausal women for femoral neck DXA-determined osteoporosis (the cut-off value of OSTA and QUS SI are -1 and 75.5). This means that QUS outperformed OSTA in primary osteoporosis screening of Taiwanese postmenopausal women under WHO osteoporosis definition and the capability of OSTA to detect primary osteoporosis in Taiwanese postmenopausal women was comparable to QUS for femoral neck DXA-determined osteoporosis.

The QUS and OSTA had better moderate to high AUC and sensitivity, and extremely high NPV for identifying middle-aged and elderly postmenopausal women at the risk of primary osteoporosis as defined by DXA at the proximal femoral site rather than the lumbar spine site. Kung AW et al. and Trimpou, Penelope, et al. conducted similar results as this study (17, 27). Chen et al. found that the r_s_ between QUS or OSTA and femoral neck BMD are better than between either and lumbar BMD (16). The above findings suggest that OSTA and QUS may predict primary osteoporosis risk in postmenopausal women more reliably at proximal femoral BMD rather than lumbar BMD. The reasons may be that lumbar BMD is affected by vertebral fractures, degenerative changes with osteophyte formation, calcification of the anterior longitudinal ligament, hyperostosis, kyphosis, intervertebral disc calcification, vascular calcification, and abdominal aortic calcification when measured by the DXA method, and its bone density may not decrease with age (28).

After further age-stratified analysis, whether osteoporosis is diagnosed according to WHO definition, femoral neck, total hip, or lumbar spine DXA T-score ≤ -2.5, QUS is more effective for osteoporosis screening in women aged 45 to 65 with better NPV than OSTA. OSTA is more effective for osteoporosis screening in women aged 66 to 80 with better NPV than QUS. Chen et al. showed that OSTA outperformed QUS with better AUC, sensitivity, NPV, and r_s_ in Taiwanese postmenopausal women over 60 years old for femoral neck DXA-determined osteoporosis (16) which is similar to and echoes the statistical results of women aged 66 - 80 in this study. QUS and OSTA indices were significantly correlated with DXA in the research and previous studies (8, 16, 17, 27, 29–32). Soft tissues and edema at the heel can artificially reduce the transmission of ultrasound across the calcaneus. Furthermore, the SI of QUS is influenced by skeletal microstructures and bone strength, which DXA does not capture (11). Persistent swelling in feet or ankles particularly over 60 - 70 years old (33). These factors may weaken the agreement between QUS and DXA or QUS and OSTA, particularly in individuals over 60 - 70 years old.

The strengths of this study include a larger participant pool than most surveys of Taiwanese postmenopausal women, which enhances reliability (16, 34). A single ISCD-certified technician used the same DXA and QUS machines for consistent BMD and SI measurements, eliminating inter-modality and inter-operator variations. Notably, this research uses DXA as the diagnostic benchmark for osteoporosis diagnosis to assess the effectiveness of OSTA and QUS in screening Taiwanese postmenopausal women. In contrast, recent studies have mainly compared osteoporosis screening tools with osteoporosis diagnosis using QUS, rarely using DXA (16, 17, 25, 26, 34, 35).

The limitations of the study include the fact that the sample was not randomly selected. The participants were primarily recruited through the Taiwan Biobank and healthcare providers. This recruitment method may have resulted in a higher osteoporosis rate of 51.89% in this study compared to a previous investigation, which found a prevalence rate of 38.3% for osteoporosis at any site among Taiwanese women aged 50 years and older (36). However, the optimal cut-off value of the study for OSTA was established as -1 under WHO definition of osteoporosis or for lumbar DXA-determined osteoporosis. This aligns with the original formula proposed by Koh et al. (8). Notably, the study identified an optimal cut-off value of 79.5 for QUS SI under the WHO definition of osteoporosis, which is close to the 78 reported by Hans et al. (37); the optimal cut-off value of QUS SI for femoral neck DXA-determined osteoporosis was set at 75.5, which is similar to the 75.7 found by Kung et al. (17). Additionally, the study is a cross-sectional study; whether the OSTA or QUS could predict the future fracture risk of Taiwanese postmenopausal women needs further prospective cohort investigation. Finally, the study is limited to Taiwanese subjects geographically.

## 5 Conclusion

Compared to DXA, QUS and OSTA are radiation-free, portable, less expensive and time-consuming, and effective clinical risk assessment tools for detecting primary osteoporosis in Taiwanese postmenopausal women.

The study disclosed that OSTA and QUS may predict primary osteoporosis risk in Taiwanese postmenopausal women more reliably at proximal femoral BMD rather than lumbar BMD. Both could independently screen and track the women at primary osteoporosis risk but not replace DXA for osteoporosis diagnosis. However, they couldn’t screen and track the women at primary osteoporosis risk interchangeably because their agreement is minimal.

For primary osteoporosis screening of Taiwanese postmenopausal women in this study, QUS outperformed OSTA with significantly superior AUC, sensitivity, and NPV (all p<0.05) under WHO osteoporosis definition. For DXA-determined osteoporosis of total hip or lumbar spine with T-score ≤ -2.5, QUS outperformed OSTA with significantly superior AUC and specificity (all p<0.05). For femoral neck DXA-determined osteoporosis with T-score ≤ -2.5, the capability of OSTA to detect primary osteoporosis was comparable to QUS with comparable AUC (p>0.05), superior sensitivity (p<0.05), and superior NPV (p<0.05).

After further age-stratified analysis for detecting primary osteoporosis in Taiwanese postmenopausal women, whether osteoporosis is diagnosed according to WHO definition, femoral neck, total hip, or lumbar spine DXA T-score ≤ -2.5, QUS outperformed OSTA with superior sensitivity and NPV in those aged 45 - 65 (all p<0.05, except for femoral neck and total hip with p>0.05), while OSTA outperformed QUS with superior sensitivity and NPV in those aged 66 - 80 (all p<0.05, except NPV under WHO definition with p>0.05). According to the above finding, DXA scanning was suggested to confirm osteoporosis if the participant has an osteoporosis result after OSTA or QUS screening.

Whether OSTA or QUS could predict the future fracture risk of postmenopausal women needs further large-scale prospective cohort investigation, categorized more finely by age group.

## Data Availability

The raw data supporting the conclusions of this article will be made available by the authors, without undue reservation.
